# MAPI: towards the integrated exploitation of bioinformatics Web Services

**DOI:** 10.1186/1471-2105-12-419

**Published:** 2011-10-27

**Authors:** Sergio Ramirez, Johan Karlsson, Oswaldo Trelles

**Affiliations:** 1 Computer Architecture Department, University of Malaga, Campus de Teatinos, 29071, Málaga, Spain; 2 Fundación IMABIS, Hospital Carlos Haya, Avda. Carlos Haya (Hospital), 29010, Malaga, Spain; 3 Instituto Nacional de Bioinformatica; Melchor Fernández Almagro 6, 28029 Madrid, Spain

## Abstract

**Background:**

Bioinformatics is commonly featured as a well assorted list of available web resources. Although diversity of services is positive in general, the proliferation of tools, their dispersion and heterogeneity complicate the integrated exploitation of such data processing capacity.

**Results:**

To facilitate the construction of software clients and make integrated use of this variety of tools, we present a modular programmatic application interface (*MAPI*) that provides the necessary functionality for uniform representation of Web Services metadata descriptors including their management and invocation protocols of the services which they represent. This document describes the main functionality of the framework and how it can be used to facilitate the deployment of new software under a unified structure of bioinformatics Web Services. A notable feature of *MAPI *is the modular organization of the functionality into different modules associated with specific tasks. This means that only the modules needed for the client have to be installed, and that the module functionality can be extended without the need for re-writing the software client.

**Conclusions:**

The potential utility and versatility of the software library has been demonstrated by the implementation of several currently available clients that cover different aspects of integrated data processing, ranging from service discovery to service invocation with advanced features such as workflows composition and asynchronous services calls to multiple types of Web Services including those registered in repositories (e.g. *GRID*-based, SOAP, *BioMOBY*, *R-bioconductor*, and others).

## Background

The Web has become one of the most important sources of information, opening access to a vast range of research resources hosted throughout the world. Unfortunately, for bioinformatics resources at least, this pleasant vision of the Web as a gallery of resources that can be discovered, combined and exploited to enhance our capacity to produce new knowledge, has a dark side. The current situation of bioinformatics services reflects a structure-chaotic set of resources, publicly available, but through a variety of mechanisms. In practical terms, each institution deploys software using its own access and invocations mechanisms and interfaces. Not only do the final users need to learn how to use the different services interfaces, but software developers spend their resources adjusting formats and protocols when combining different services.

The most promising solution for these problems is based on the notion of Web Services (WS). WS are applications deployed over the Internet providing a given functionality, to other applications. WS technology resides in a stack of *XML*-based protocols such as *WSDL *(Web Service Description Language [1,2]) and *SOAP *(Simple Object Access Protocol [3,4]) to allow automatic access to software running on different platforms and implemented in different programming languages. WS differ from Web applications in that they generally involve application-to-application communication, and are not intended to be accessed via a Web browser. Instead, clients must be written in a language that supports *HTTP *and *SOAP*, and issue a message or remote method call a WS, etc, which in turn processes the message and returns a response to the client. In the context of bioinformatics, Web services promises easier integration and interoperability between bioinformatics applications.

However, even when WS represent a step forward in process intercommunication, it is not mandatory to use *SOAP *in the WS implementation. For example, other WS use *XML-RPC *[5] based on a simpler communication mechanism although it does not cover all *SOAP *functionality; and *REST *[6] does not define the structure, composition or content of the data interchanged. To overcome this problem, the bioinformatics provider community has proposed complementary solutions.

One specialized approach which aims to improve metadata service annotation in bioinformatics is *BioMOBY *[7]. This standard aims to facilitate the integration of services by using a metadata repository (*MOBY Centra*l [8]) with service descriptions defined using a service ontology. In this system, all the services use the same format, and so the outputs can be directly connected to other services.

One example of a successful service repository in bioinformatics is the repository for BioMOBY services. A repository stores definitions of WS entities and related objects (e.g. I/O specifications), but does not usually provide more functionality beyond making the discovering of new services easier. Clients are still responsible for tasks such as processing the inputs to meet the needs of each service.

In this sense, data integration in bioinformatics remains a problem, partly because many services declare inputs and outputs as plain strings. In the discussion section, we will give an example of how this can produce problems when trying to integrate services.

Therefore, the problem of service integration and interoperability remains unsolved and it is not difficult to predict that new protocols are still appearing.

As a contribution towards solving the limitations of working over specific repositories and service technologies, we have developed *MAPI*, which is a programmatic framework that integrates different repositories schemas through a virtual definition and can be easily extended to include different Web Service invocation protocols.

The versatility and strength of *MAPI *has been demonstrated by the implementation of different clients such as *JORCA *[9], and *Magallanes *[10] which are able to access repositories such as *MOBY Central *at the *University of Calgary *that uses the *BioMOBY*-based repository http://moby.ucalgary.ca/moby/MOBY-Central.pl, the National Institute for Bioinformatics in Spain which uses an extended *BioMOBY *repository http://www.inab.org/MOWServ, the tool-metadata repository used in the *Advancing Clinico Genomic Trials EU-project *http://www.eu-acgt.org/, *EBI *web services [11], *Biocatalogue *[12], *EMBRACE *[13], *WABI-Japan *[14] and others.

## Implementation

### Modularity

**Figure 1:**
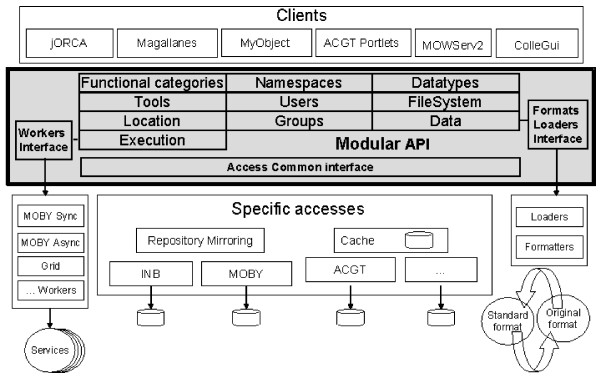
**The most important MAPI modules and their dependencies**. The core module allows the definition of available data types in the system. Two main branches of dependencies correspond to Tools (software components that consume and/or produce data) and handling of a particular data type (Data). The Execution Module combines these two branches by sending the data to the tools for execution using the information stored in the ToolLocation module. The modules for NameSpaces and Users are not shown for simplicity since they can be used with all the other modules.

One of the most relevant challenges that the developers have to face when developing software clients is the diversity of types, such as clients, browsers, file explorers, execution systems and catalogues. This perception has driven the modular design of *MAPI *(see Figure 1); gathering in each module a closely related set of capabilities, e.g. service handling, execution, data conversion, security. One benefit is to install exclusively the modules the programmer needs (proportionality) for each specific environment, avoiding the full library. The core modules of MAPI manage the execution of distributed services, including: handling Tools (Services, Applications and Workflows) and their functional categories or User Data.

### General functionality

A core set of functions is always attached to each module to support retrieval, query, editing and management of the specific resources the module handles. Consequently, the module related with data types is able to retrieve I/O compatible services, the user module provides privacy and user authentication; the tasks module can invoke services, etcetera. Worthy of mention are the "*Filter Lists*", which modules return. The contents of these lists can vary depending on the filters defined by the software developers, providing an extensible and customizable method to query the data sources. This is more flexible than platforms which only provide fixed and pre-established searches.

All MAPI components are available to system developers through a configuration file, yielding the access to multiple data sources without any change in the application (Client) code. Two different configuration levels can be devised related to "where and how" the information is. That is to say, the client can switch between BioMOBY-like repositories wherever it is located, but also can switch to work with *WSDL *definition of WS, such as the *WABI *collection. In the line of customization, the configuration file can also be used to define the behaviour of each module to fit specific requirements. In this sense, it is possible to define some aspects such as delayed writing, the usage of a cache system or the possibility of modifying the information.

### Accesses

In order to make each module independent of external resources and their internal organization, each module has a component, called Access, that defines the metadata resources and how they will be accessed, allocated and connected according to the module functionality. Therefore, one of the most important tasks covered by the Access components is the mapping of WS description into the internal data model used by *MAPI*.

The content of the internal data model was decided by an exhaustive review of current implementations, such as BioMOBY, WSDL, Grid Systems, etc. Naturally, this data model can also become incomplete or obsolete when new functionalities offered by WS would need additional descriptors. To prevent against this problem, we devise three different ways for rapid extension of functionality by: (a) adding new modules with the new functionality (e.g. for the management of "groups" of users); (b) for existing modules, the extension is accomplished by inheritance of the basic functionality and incorporating new methods, attributes, information, etc.; and (c) the resource's attributes in any module can be extended by adding 'type-label-value' fields (e.g. the location modules need different fields for different protocols: *BioMOBY *needs the *URL*; *SOAP *Web Services requires *URL *and namespace; *Grid *services need authentication information, etc).

At the same time that *MAPI *can be extended to cover new aspects in integration, it is imperative to ensure backward compatibility, that is to say, existing clients will continue to be valid, and new clients would exploit the new functionality.

### Workers

In the same way as the different descriptions of resources are homogenized by Access modules, we also have to take care of the different invocation methods for WS New simple pieces of software named *Workers *performing the uniform invocation of services. In this case, different workers can be plugged into the system and therefore the system is able to invoke services with different calling protocol (in the same session). This ability makes *MAPI *suitable to run different types of services, which allows the creation of new and more complex workflows, for instance to be able to combine BioMOBY and EBI services. In this way, in order to make it possible to launch new types of services, such as REST, for example, only the implementation of a new *Worker *is necessary.

The specific task of the workers is to deal with the different invocation protocols and formats in which the data can be coded. The latter is provided by the data module which is able to handle a set of plug-ins (named *Formatters*) aimed at translating the data from their native format to the generic one expected by the *Workers*.

**Figure 2:**
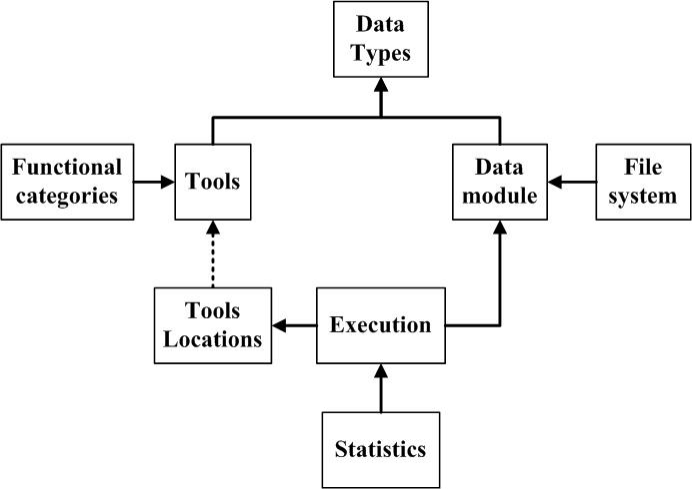
**Full diagram of the framework developed**. The figure shows the different software components that comprise the overall system. Clients access global functionality using only those modules that handle the resources needed. Between the modules and the original data repository there are specific accesses. Other accesses complete the functionality for Repository Mirroring, Cache. The Workers, Formatters and Loaders (on the sides) complete the system, enabling the access to different service protocols and data formats.

In Figure 2, all elements described in this section are shown to give an overview of the *MAPI *architecture.

## Results

For more in-depth exploration of *MAPI*'s capabilities, we will walk you through the typical developer steps when building integration software. Detailed information on this exercise is available in the Supplementary Material (see Additional file 1) and comprehensive material for training is available on the *MAPI *web page.

### Repository homogenization

The first integration requirement is to resolve heterogeneity in the web services definition. This is achieved through Access, which recovers all necessary functionality from the specific repositories. Thanks to this, in *MAPI*, the type of system, the specific functionality provided or the way to access to the information are not important. The developer only has to learn how to use a single interface to access all these systems.

Typically, writing an *Access *will demand the mapping of the original data model into the one used by *MAPI*. This task may require different times, depending on the differences between the modules, for example it took one person one week to complete the mapping of *WSDL *services (i.e. writing one tool *Access *for WSDL service information and another for XML Schema). It is noteworthy to observe that once the code for accessing information in WSDL descriptions has been developed, any WS described using WSDL will be accessible via MAPI. For example, we are able to access web-services from European Bioinformatics Institute (EBI) and the Web API for Biology (WABI) using the same accesses.

The access implementation uses the API of the resource in question. For example, if the API of the BioMOBY registry (MOBYCentral) changes, it is obviously necessary to modify also the access code. However, in general, software API specifications are stable and changes are minor. So far, we have not needed to do major rewrites of the access code on account of changes in repository APIs.

The developers can choose the access to use by using the function ToolModule tools = new ToolModule ("X"), where "X" is the path to the configuration file which defines the repository for use. At this point, the differences between repositories are hidden, and the developers can query the information in the same way, independently of the type of repository.

Currently, *MAPI *is able to integrate the following repositories:

• Several *BioMOBY *repositories (Central at http://moby.ucalgary.ca, the IRRIA web services at http://cropwiki.irri.org)

• The National Institute for Bioinformatics Spain at http://www.inab.org/MOWServ using an extended version of *BioMOBY *central system, with additional documentation and input/output examples.

• the *ACGT Grid *services developed in the framework of the *ACGT *project at http://www.bitlab-es.com/ACGTRepository

• *WABI *Japanese web services (Web API for Biology) at http://xml.nig.ac.jp/index.html

• *EBI WSDL *services at http://www.ebi.ac.uk/Tools/webservices

### Finding the right resource

Browsing and searching for resources, such as services, are typical functionalities offered by Clients. *MAPI *provides multiple supports for querying the metadata to identify the right resource. In this way, it is possible to search, for example, by identifier (getTool, getTools), by name (searchTool) or even create customized filters to fit the requirements of each Client:

Tool tool = MOBY_Tools.getTool("id");//Search by id

Tool tool = MOBY_Tools.searchTool("name");//Search by name

FilterList<Tool> tools = MOBY_Tools.getToolList();//Custom filters

tools.addFilter(new Filter<Tool>() {

   public boolean test(Tool element) {

      return element.getName().startsWith("analyze");

   }

});

tools.clearFilters();//remove the filters

As can be seen in the code, the first lines show how to retrieve tools by 'id' or by 'name' (general functions) and also how to create a customized filter; in this case to return all the tools whose name begins with "analyze".

The power of these simple functions becomes evident when Clients implement complex search algorithms based on them. For instance, *Magallanes*; an application for the discovery of services and datatypes, uses the primitives provided by *MAPI *to develop *Google-style *'*did you mean?*' methods.

Another service that is increasingly in demand is the 'searching for compatible services' (pipelining of services through the Input-Output). To create a workflow it is necessary to find those tools which accept the output from another tool as input. MAPI provides the searchToolsCompatibleWith function, that returns a list of Tools, compatible directly (child datatypes) or indirectly (all the descendants) with a given Datatype. For instance, *Magallanes *creates workflows automatically, based on the initial and the final datatypes of the workflow. This feature is also used by *jORCA *to provide the user with a list of the Tools that can accept a specific file as input data.

### Invoking tools

Heterogeneity is not only a problem of repositories, but it is also present in the service's invocation procedures. The execution module -that contains the mechanisms to invoke the tools defined in the Tools module- includes the addTask function to create a new task associated to the appropriate worker (specific for each invocation protocol).

Using a simple function such as Task t1 = em.addTask(tlm.getTool("toolID"), input, output) the developer does not have to worry about the type of service or the underlying protocol. Clients such *jORCA *exploit this functionality.

### Handling data

However the homogeneous invocation of tools is not enough to ensure tool integration, since services may require data in different formats. MAPI handles user data with a common model, regardless of the format in which they come.

The Data module -in charge of the uniform handling of datatypes- provides the functions to change the format of data. Since these functions are coded in the Formatters, the ability to extend the scope of data translation is always available. Functions such as 'Data data = dataModule.newData(file, source)' are able to understand the data stored in a file in a given format and transform the same data into a new target format using data.getRawContent(target). Using this strategy, the format of data can be modified as it is needed by the service to be invoked.

In the cases in which the source format is unknown, MAPI has also "Heuristics" components which include different algorithms for managing in automatic way the format of the information [15].

Data integration in bioinformatics remains a problem, partly because many services declare inputs and outputs as plain strings. In our opinion, the use of structured information should be promoted, to simplify service integration.

### Cache

In the management of remote services, care must be taken not only with the availability of the repositories, but also in the response time of the Web. A cache is a local copy of metadata that prevents against repositories' availability and seriously reduces starting time and speeds up access.

The configuration file is used to specify if a cache is used or not. This means that the same source code can be used in either case.

## Discussion

In this section, the scope and efficiency of MAPI is compared against different solutions for the integration of Internet resources in the field of bioinformatics.

BioBroker [16] makes use of mediators to build up a uniform view of data from different sources. This program can be extended to use new data sources and programs, but its rigid architecture and built-in definitions do not allow the addition of new types of resource handlers.

The MyGrid [17] project provides a technology stack for service oriented architectures with support for essential tasks such as workflow editing and enactment (Taverna [18]), workflow sharing (myExperiment) and service registration (BioCatalogue). Taverna is able to create workflows using services from multiples sources; but the user has to provide an additional code to convert the structure and format of the data returned from a service into the format required by the next service in the workflow. This is similar to formatters, but in MAPI, they are used automatically without user intervention.

BioMOBY offers a solution to the integration of services, based on the use of a taxonomy of data types, shared by all the services registered on the same server, which supports the identification of compatible services and their interoperability. These solutions provide a mechanism for the rapid discovery of services, but do not support other kinds of resources or extend to other kinds of services.

On the other hand, WSMX [19] allows the transparent execution of different types of services and homogeneous access to data. However, WSMX is limited to web-based technology and is consequently unable to combine services implemented with other technologies such as Grid Services, local scripts, etc. unless a WSDL definition has been provided.

Finally we must talk about Cactus [20]. This framework is quite similar to MAPI, offering functional modularity, configuration options and access to multiples data sources. However, each change in the configuration implies a re-compilation of the program and, with it, the need to distribute the application source code, which may not be possible in all situations. Furthermore, unlike MAPI, the framework does not show a strong architecture that allows identifying the components and the dependencies among them.

As we can see, these solutions offer so many or so few features focusing on solving very specific problems. They do not provide the flexibility and extension features than the modular architecture of MAPI. Also, MAPI does not force the use of any format or particular data source, and the developer can adapt the behaviour of each independent module to his particular requirements and combine them in multiple ways to obtain the desired functionality.

In any case, MAPI does not solve all of the problems deriving from heterogeneity. One important limitation is due to the existence of unilateral formats. Some formats lose information during the transformation process. This means that the original format cannot be recovered, at least in its entirety. Take for example the case in which a sequence is extracted from a GenBank [21] file. We can obtain the sequence, but cannot retrieve the entire file because the rest of the information is lost. For further details, please see the additional material where we have included a detailed example to illustrate the concept of data mapping in MAPI.

Another example is the case of the use of different datatypes structures in several repositories. All the compatibility functions are based on the existence of two resources which share the same datatype; but identifying two different datatypes as the same one is no easy task. Features such as the name or the identifier can vary through multiples sources and have different meanings. For example, a sequence is not the same in biology (amino acid sequence) as in multimedia (video sequences). In the same way, neither is the structure of a datatype an identifying element. Each user could define every entity with a different datatype; this does not have to agree with that of another user. Moreover, two datatypes with an identical structure could represent two completely different entities.

A practical example which illustrates the need for structured information and semantic descriptions of services is the set of services from WABI. Those services declare strings as inputs and output and only provide human-readable descriptions. After a careful analysis, we mapped, by hand, the inputs/outputs of those services to the structured data model used in MAPI. In our opinion, if the service provider maps/links the service inputs/outputs with semantic information, service integration is greatly simplified.

In all of these situations, human intervention is needed in order to identify the similarities. MAPI provides mechanisms (called Loaders) to integrate two repositories once the differences have been determined. These mechanisms allow changing the structure of a datatype with the purpose of fitting user requirements. For example, hiding some attributes which can be calculated from others, or more importantly, changing the structure of one datatype to fit that of another.

The current version of MAPI is more focused on reducing the problems with syntactic heterogeneity. The semantic heterogeneity is addressed manually in the implementation of Access and Formatters, by for example mapping the information to the appropriate fields in the data model used in MAPI or by loading the information to the correct structure.

The support for semantics is limited in MAPI at this moment but we plan to develop new modules to express semantic relationships between the metadata used in MAPI.

Despite these limitations, MAPI provides a flexible and adaptable solution to most problems arising from information heterogeneity.

## Conclusions

Considering the diversity and multitude of web services for bioinformatics, it is becoming increasingly complicated to develop efficient and generic client software to take advantage of these important web resources. The reason is that these web services are made available using different web-service styles and consume/produce different data formats. Moreover, metadata for discovering and exploiting web services are registered in different types of metadata repositories. In this scenario, the need for a unifying software framework for client development is clear.

This paper reports a software framework, called MAPI, which aims specifically at simplifying client software development in bioinformatics. The framework provides functionality to connect to various metadata repositories, invoke web tools and translate between various data formats.

Client development is simplified since MAPI provides a unified view of different web resources (services, repositories, data etc). By dividing functionality in modules, it is also possible to combine these modules to only include the functionality required by the client in question.

Each module is divided into several layers: the top layer, which is the layer which software developers use, and one or more access layers, which contain the specific instructions for accessing the web-resource (for example a metadata repository etc). In some cases, the access layer uses other components. For example, communication with web services is accomplished through software components called workers and data format translation is done using formatters.

MAPI thereby provides a stable base for developing client software (i.e. developers use the top layer) which, at the same time, can be extended to deal with new service protocols or metadata repositories (by adding new workers or accesses). The framework is complete in the sense that developers obtain abstractions of all crucial client-related tasks when using bioinformatics services. Although other, specialized solutions are available which, in some senses, provide more advanced functionality than the current implementation of MAPI does, it is important to note that the top layer is abstract, meaning that the underlying software code, which performs the actual work (reading metadata from various repositories, caching data, executing services etc) can, in most cases, be modified or even completely replaced with improved versions without affecting top level client software.

The support for several existing web service architectures is included by default within the framework. This allows client software to discover resources and invoke web-services from several important architectures in bioinformatics, including BioMOBY, simple SOAP-services described using WSDL and Grid-services (specifically in the ACGT project). A clear indication of the usefulness of mAPI is that it has already been used as a basis for developing several novel clients in bioinformatics; see for example jORCA, Magallanes and the ACGT grid architecture.

In summary, we believe that this software framework is an important step towards the integrated exploitation of bioinformatics web services.

## Availability and requirements

MAPI, including the complete installation procedures, user manuals and tutorials is freely available at http://chirimoyo.ac.uma.es/mapi/ and http://www.bitlab-es.com/mapi/

The code requires Java version 6 or above to compile.

## Authors' contributions

SR has designed the complete MAPI framework and has implemented the execution module and the workers. JK has advised on MAPI design and coordinated ACGT Implementation. OT has coordinated and organized the entire development process. He has support the development by providing ideas and making development decisions. All authors have read, participated in and approved the final manuscript.

## Supplementary Material

Additional file 1 **Supplementary material**. The file contains extended explanation of the features that *MAPI *provides including schemas about how the information is modelled, code examples and a section answering the most frequently asked questions. Click here for file
