# Supplementary material for: MAPI: towards the integrated exploitation of bioinformatics Web Services
Source: BMC Bioinformatics. 2011 Oct 27;12:419. doi: 10.1186/1471-2105-12-419 (PMC3219744; doi:10.1186/1471-2105-12-419)
Supplement: Additional file 1 — Supplementary material. The file contains extended explanation of the features that MAPI provides including schemas about how the information is modelled, code examples and a section answering the most frequently asked questions. [file 1471-2105-12-419-S1.PDF]

Supplementary Material

MAPI: towards the integrated exploitation of bioinformatics Web Services

Sergio Ramirez, Johan Karlsson and Oswaldo Trelles.

October 3, 2011

Contents

1 Internal data models **2**

2 Example of information mapping **7**

3 *MAPI* usage example **8**

4 FAQS **10**

1 Internal data models

This section will describe the different data models in *MAPI* for representing information about services, datatypes etc.

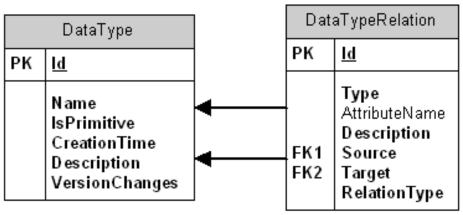

Figure 1: Model for data types. As can be seen, the basic concept is the DataType with name and description etc. Relations between data types (the structure of the taxonomy) are modelled by the DataTypeRelation concept.

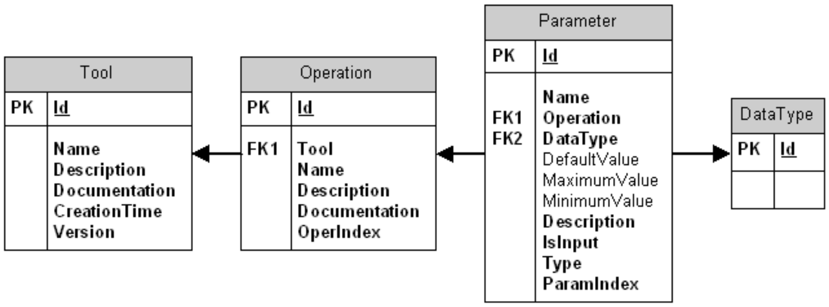

Figure 2: Model for tools. In this case the main concept is Tool, which consists of a set of operations, which, in turn, has a set of parameters. In the case of the parameters, it is possible to represent if the parameter accepts single or multiple values and the maximum or minimum values. Finally, we can note that the relationship between this module and the data type module is established through the operation parameters which indicate the type of data for the input/output parameters.

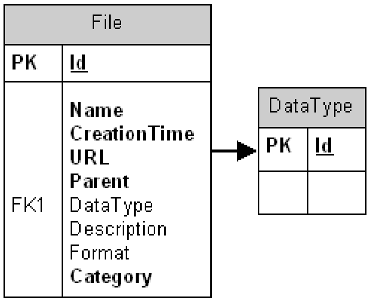

Figure 3: Model for files. As shown in the figure, the model consists of a single table to model two concepts (files and directories). The field category is used to differentiate between these types. For both resources, we can specify attributes such as name or date of creation, and most importantly, the directory where the file or directory is located, with the attribute *Parent*.

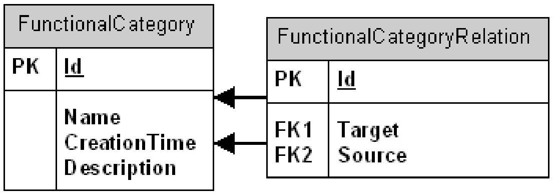

Figure 4: Model for functional categories. This module is responsible for providing a mechanism to define functional categories which organize the different types of resources. Each category has a name, description and date of creation. It is also possible to establish relationships with other categories to organize them hierarchically. Each category can have several parents.

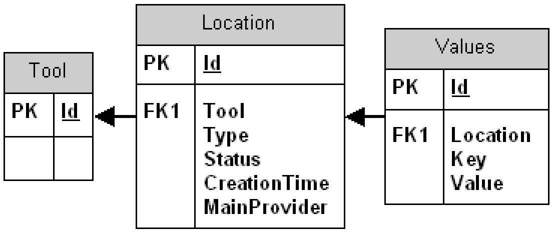

Figure 5: Model for locations. This module is focused on the management of service instances and therefore the data model has been oriented to allow the management of the diverse information in these resources. The information is organized in two main parts: one generic part with information about service instances (the table *Location*); with information such as type of tool (for example, BioMOBY service) or the creation date of the metadata. In the table *Values* it is possible to specify all values needed to invoke a specific tool. Because it is impossible to predict this (depends on the invocation protocol), the information in this table are stored as tuples of the type *key / value*.

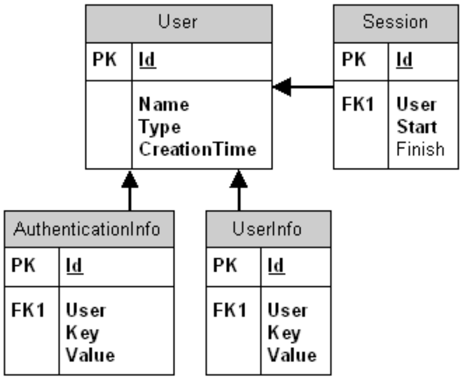

Figure 6: Model for users. Information about users is very diverse, so we have used the same solution as for locations. In this case the information has been divided into general information and information used for user identification. The model also includes information about the user creation date and type (Administrator, Registered or Temporary) and the ability to control the sessions that the user has opened and their duration.

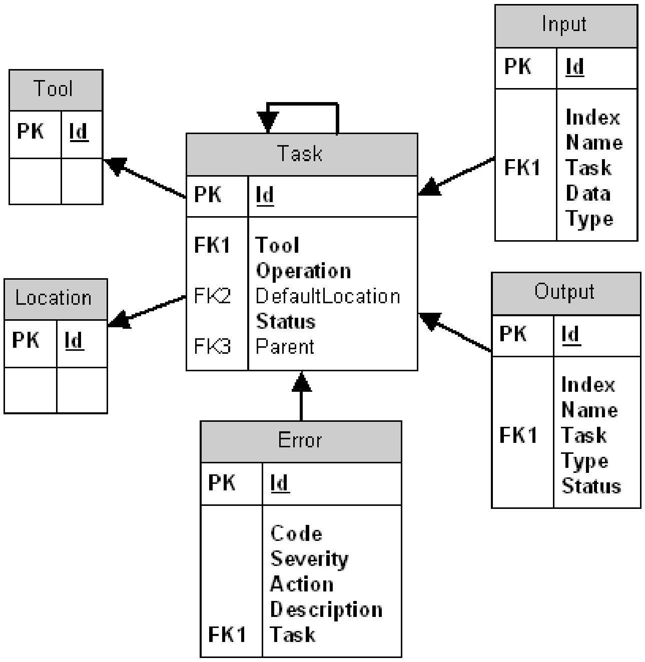

Figure 7: Model for the run time (tasks). The main focus in this model is the tasks, representing the processes running. The tasks indicate the tool and operation invoked and the current state of the task. Tasks can produce error messages which are represented in the error table, with error code, a description of the error occurred, the severity (*Error* - total failure or *Warning* - partial results produced).

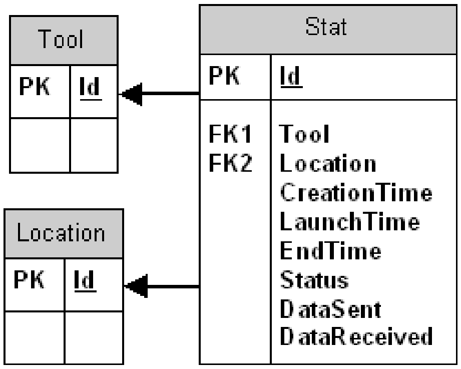

Figure 8: Model for statistics. This module will model the statistics produced by the execution of different tasks. The model represents which tool was invoked, which location, time of task creation/launching and completion. It is also possible to represent the size of data sent and returned.

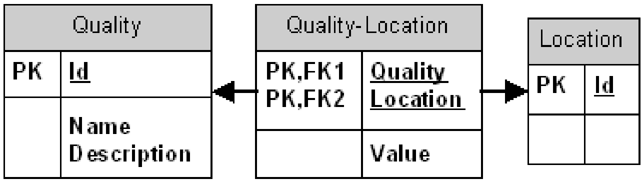

Figure 9: Model for tool location qualities. This model can extend the information in the model for location with information about features such as asynchronous calls supported, error control or data persistence mechanism. This consists of a feature name and a brief description.

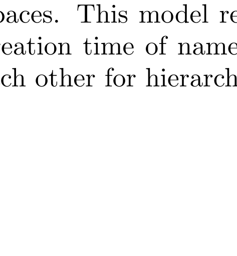

Figure 10: Model for namespaces. This model represents information such as the name, description and creation time of namespaces. It is also possible to associate namespaces with each other for hierarchical organization.

2 Example of information mapping

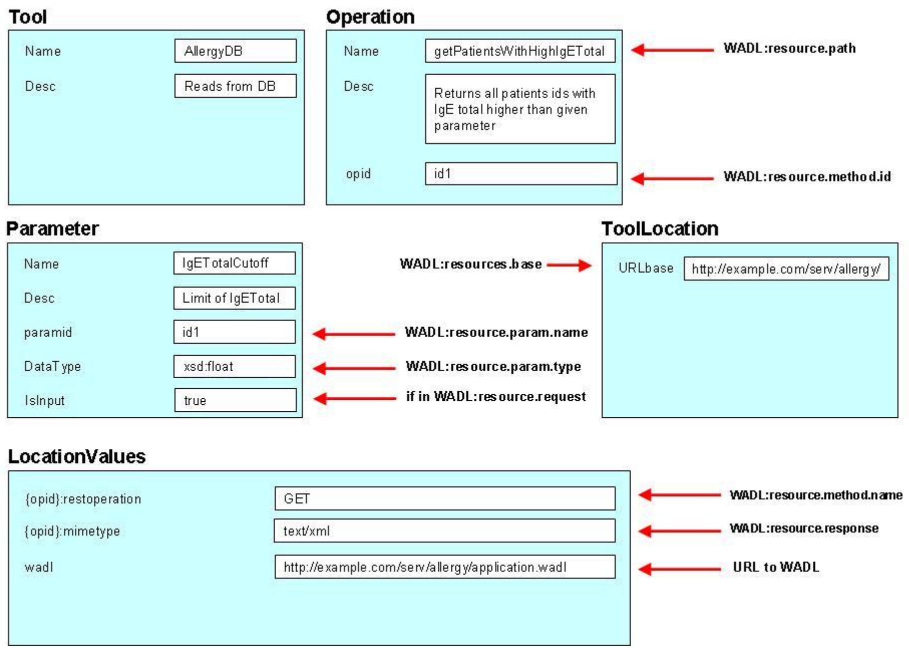

Figure 11: The *Accesses* are one of the most important pieces of *MAPI*, since they map the information from the original data model to the internal data models used in *MAPI*. In this figure, we show how elements in WADL descriptions of RESTful services can be mapped to the data models in *MAPI*.

3 *MAPI* usage example

MAPI has a large and rich set of functions available for the developers and sometimes it can be difficult to know which modules to include or which methods to use. We exemplify usage of *MAPI* with a small example of loading a genetic sequence from a file, manipulate the information, store in other formats and, finally, search for compatible tools.

Code 1 Creating module instances. As a first step, we have to create the modules that we are going to use. For now we are going only to manipulate user data files and we only need to create instances of the data and dataype modules. Creating module instances consist of indicating the file that contains the module configuration and, when necessary, include instances of other modules the module in question depends on (only the data module in this case).

```
DataTypeModule dataTypeModule = new DataTypeModule("conf/
    api.chiri.conf");
DataModule dataModule = new DataModule("conf/api.chiri.
    conf", dataTypeModule);
...
```

Code 2 Loading a sequence stored in the Fasta format. Note that when the format is known, it is only necessary to indicate the format and the file that contains the sequence to load it into a manipulable data instance.

```
...
Format source = dataModule.getFormatsByName("Fasta").get
    (0);

Reader file = new BufferedReader(new FileReader("filename"
    ));
ComposedData data = (ComposedData)dataModule.newData(file ,
    source);
...
```

Code 3 Accessing a data instance. Once the information has been loaded, *MAPI* provides a complete set of functions to manipulate and access the information. It is possible, for example, to list the attributes of a structured data or to read the values of specific attributes.

```
for (String attribute : data.getAttributes().keySet())
  System.out.println(attribute);

BasicData SequenceAttribute = (BasicData) data.getData("
    sequence");
BasicData LengthAttribute = (BasicData) data.getData("
    length");

System.out.println("Sequence: " + SequenceAttribute.
    getValue() + ", length: " + LengthAttribute.getValue()
    );
```

Code 4 Transforming a data instance. With the loaded data instance, it is also possible to convert the information in others formats (listed in the *MAPI* configuration file). Simply indicate in which format do you want to convert the information. *MAPI* can also list the formats compatible with the datatype of the loaded data.

```
...
for(Format target : data.getCompatibleFormats())
{
  System.out.println(data.getRawContent(target));
  System.out.println();
  System.out.println();
}
...
```

Code 5 Searching for compatible services. We have seen how data can be loaded, manipulated and transformed. Now, we want to discover what we can do with the data, i.e. which tools are compatible with the data.

```
ToolModule ToolModule = new ToolModule("conf/api.chiri.
    conf", dataTypeModule);

boolean exact = false;
FilterList<Tool> compatibleTools = ToolModule.
    searchToolsCompatibleWith(data.getDataType(), exact);

for(Tool tool : compatibleTools) System.out.println(tool.
    getName());
```

4 FAQS

What kind of effort is required to add a new repository? *MAPI* requires the development of an access to read, write and map the information of a specific type of resource onto the unified model. Examples of resources include repositories, services, data-types etc. However, it is important to note that the same access is used for all instances of the type of resource it represents.

For example, this means that once the code for accessing information in WSDL descriptions has been developed, any WS described using WSDL will be accessible via *MAPI*. Note that, since *MAPI* splits the service and data type metadata in two different modules, it was necessary to write two accesses, one for mapping the service descriptions and one for the datatypes (XML Schema). For instance, the same two accesses are used to parse/map WSDL descriptions of services from the European Bioinformatics Institute (35 services) and from the WABI (21 services).

What happens when a repository is updated? how is the new mapping added? Regarding repositories: WSDL descriptions typically only describe a small set of services. It is therefore good practice to register service descriptions in a shared service metadata repository (such as the repositories in BioMOBY). The strategy of re-using accesses is also followed for BioMOBY repositories: only three accesses (for tools, data types, functional categories respectively) were necessary to develop. Once developed, any BioMOBY service repository instance (main repository in Canada, INB repositories in Spain, etc.) can be accessed using the same accesses.

Regarding the repository update model:

1. If new service metadata are registered in the repository or if the description of existing services is modified, nothing in the access needs to be changed. However, special care must be taken when client software is using the built-in cache system in MAPI. The use of the cache system is configurable for each module. The client software can request to the cache system to update the metadata either directly (one-time refresh) or configure the cache system to automatically refresh.

2. The access implementation uses the API of the resource in question. For example, if the API of the BioMOBY registry (MOBYCentral) changes, it is obviously necessary to modify also the access code. However, in general, software API specifications are stable and changes are minor. So far, we have not needed to do major rewrites of the access code on account of changes in repository APIs. In the case of WSDL services, it is conceivable that the WSDL specification could change (i.e. a new version) and therefore make it necessary to rewrite the access. However, considering that it took approximately one week to develop this access from scratch, this is a minor task.

3. Noteworthy to observe, is that existing clients do not need to modify / adapt their code, at least they are going to use the new fields of the updated version. In this sense, downgrade compatibility is offered by *MAPI*.

How many format adapters do you (or a third party programmer?) need to write? is there a common target format? The strategy in *MAPI* is similar to shims in Taverna. The advantage of *MAPI* compared to Taverna, is that these conversions are done automatically by the system when *MAPI* discovers that the services cannot be directly glued together without format conversion. *MAPI* can access a common model for user data (via the datatype module). It is possible to write a formatter (one per format), which maps the data onto this shared data model. For example, the internal data model used by default in *MAPI* for sequences consists of sequence data and length of sequence. We have implemented several formatters, one of those formatters maps a FASTA formatted data onto this internal data model for sequences. We have also done a formatter for BioMOBY formatted sequences. This means that user data formatted as BioMOBY is mapped without loss against the internal data model. FASTA sequences contain the sequence data itself but not the length. So, in our example of connecting a service which outputs FASTA with a service that requires as input BioMOBY sequence, the following steps would happen:

1. *MAPI* recognizes that a service that expects BioMOBY formatted sequence is connected with user data formatted as FASTA.

2. *MAPI* dynamically loads the formatter for FASTA and invokes the formatter with the user FASTA-formatted data.

3. The formatter loads the user data into main memory and the length of the sequence data is calculated to complete the required internal model for sequences.

4. *MAPI* loads the formatter for BioMOBY sequences and notifies it to write the sequence data in BioMOBY format.

In our work Biodata-SF (Nettab 2009), we describe in detail how to perform data transformation based on the *MAPI* approach. Additionally in the Supplementary Material, we include a simple example of a program that is able to read a file in a certain format and show it in another format. The user specifies the format of the input file and the requested output-format. Note that the *MAPI* architecture also includes heuristics, which can help when the user is not sure of the input format.

How MAPI handles the user data heterogeneity? Our approach tries to make simpler to address the problem of heterogeneity by providing different tools to manage it:

1. A set of "heuristics" plugging -which is extensible- to allow the automatic recognition of a file format. The number of heuristics that can be plugged into the system is not limited, and the user is able to specify the more appropriated heuristics to satisfy the requirements. For example we have some implementations of heuristics based on rules and regular expressions (see our work Biodata- SF; Nettab 2009), which can recognize biological sequences in different formats. Other rules can be used to recognize other text formats like Blast or ClustalW outputs.

2. By default, *MAPI* provides a set of formatters (also extensible) that are able to read a file in a given format and return the information in a structured way (mapped to an internal data model). Using this data structure it is easier to access the information because is organized in fields.

This is only a partial solution and we recognize that solving the data integration problem in bioinformatics is still an open problem. From our point of view, a global agreement for a solution is needed; but we believe this action is out of the scope of our manuscript.

how the approach can be extended on other possible Web Service invocation templates which could be define in the future.? This topic of the paper is service integration (web services integration, to be specific). However, we have also integrated successfully other type of "tools" such as grid services, workflows, etc. (note that grid services are implemented as SOAP services, but with authentication). For instance, the *jORCA* Client which is based on *MAPI*, has successfully integrated *Services Functional categories* or *Data Files type* of resources. It is noteworthy to observe, the integration of new types of services only requires the development of a new worker, since *MAPI* deals with all services types in an abstract way. It is not important which communication protocol that is used, as long as the service metadata can be mapped to the internal data model Other types of resources have also been integrated with *MAPI*, such as user data files and users information.
